# Extrapyramidal symptoms predict cognitive performance after first-episode psychosis

**DOI:** 10.1038/s41537-022-00270-8

**Published:** 2022-08-04

**Authors:** Maija Lindgren, Sebastian Therman, Anna Avellan, Tiina From, Jarmo Hietala, Minna Holm, Tuula Ilonen, Tuula Kieseppä, Heikki Laurikainen, Raimo K. R. Salokangas, Jaana Suvisaari

**Affiliations:** 1 Mental Health, Public Health and Welfare, Finnish Institute for Health and Welfare, Helsinki, Finland; 2 Department of Psychiatry, University of Turku and Turku University Hospital, Turku, Finland; 3 Turku PET Centre, University of Turku, Turku, Finland; 4 Department of Psychiatry, University of Helsinki and Helsinki University Hospital, Helsinki, Finland

**Keywords:** Human behaviour, Psychosis

## Abstract

Extrapyramidal (EP) symptoms such as tremor, rigidity, and bradykinesia are common side effects of most antipsychotics, and may associate with impaired performance in neurocognitive testing. We studied EP symptoms in first-episode psychosis (FEP; *n* = 113). Cognitive testing and EP symptoms (three items of the Simpson-Angus Scale) were assessed at baseline and follow-up (mean follow-up time 12 months). Mild EP symptoms were present at treatment onset in 40% of the participants. EP symptoms were related with lower performance in neurocognitive testing at baseline and at follow-up, especially among those with nonaffective psychotic disorder, and especially in tasks requiring speed of processing. No associations between EP symptoms and social cognition were detected. In linear regression models, when positive and negative symptom levels and chlorpromazine equivalents were accounted for, baseline EP symptoms were associated with worse baseline global neurocognition and visuomotor performance. Baseline EP symptoms also longitudinally predicted global, verbal, and visuomotor cognition. However, there were no cross-sectional associations between EP symptoms and cognitive performance at follow-up. In sum, we found both cross-sectional and longitudinal associations between EP symptoms and neurocognitive task performance in the early course of psychosis. Those without EP symptoms at the start of treatment had higher baseline and follow-up neurocognitive performance. Even mild EP symptoms may represent early markers of long-term neurocognitive impairment.

## Introduction

Extrapyramidal (EP) symptoms are caused by disordered dopaminergic regulation of movement, and are prototypically seen as parkinsonian symptoms, such as tremor, rigidity, and slowed movement. Mild EP symptoms are common already among individuals experiencing their first psychotic episode^1,2^, making the person appear slightly inexpressive and slow, thus overlapping with so-called negative symptoms. Most antipsychotic medications have a potential to induce EP symptoms as a side-effect^3,4^. The first generation, but also the second generation antipsychotics, are known to induce EP symptoms^5^. It has also been reported that motor impairments associate with clinical features of psychotic illness, such as negative symptoms^2^, although not all studies have found such associations^6,7^. Motor impairments may also index disease severity^8^ and predict a worse outcome of psychosis^9–11^.

Cognitive impairment is a common feature of psychotic disorders^12^. Similarly to EP symptoms, cognitive deficits associate with negative symptoms^13^, a more severe clinical picture, and worse functional outcome^14,15^. While cognitive performance may be influenced by antipsychotics^16^, the association is complex^17^.

Motor impairments have been found to be linked to greater cognitive impairment in psychotic disorders^9^. In individuals with schizophrenia, severe EP symptoms have been associated with worse neurocognitive performance when controlling for severity of psychopathology, both at a composite score level and in several cognitive domains such as verbal memory, processing speed, and working memory^18^. In another study, motor impairment predicted a cognitive factor in people with schizophrenia when age, gender, and education were controlled for^19^. In individuals with schizophrenia or schizoaffective disorder, some cognitive tests correlated with psychomotor abnormalities^20^. Neurocognitive performance has been reported to associate with EP symptoms also among outpatients with schizophrenia at baseline and 6-month follow-up, when controlling for anxiety and depression^21^. Furthermore, a recent study among people with schizophrenia found that neurocognition, but not social cognition, was directly correlated with EP symptoms^5^. Studies on the association between EP symptoms and cognition at the beginning of psychotic disorders have been scarce. Among persons with first-episode psychosis (FEP), EP signs longitudinally associated with deficits in memory, executive functioning, and attention in 6-month follow-up assessments^22^. However, not all studies have found connections between motor and cognitive impairments in FEP^6^.

EP symptoms do not seem to merely reflect medication side effects but may represent a neurobiological mechanism related to the etiology of psychosis. It has been proposed that in addition to being antipsychotic drug induced, motor impairments in psychotic disorders may be spontaneous^9^, and could thus be an intrinsic feature and a possible endophenotype of psychotic disorders, even marking liability to schizophrenia^23^. EP symptoms have been described in neuroleptic-naïve patients, unaffected first-degree relatives, and high psychosis risk individuals^18,19,24^. In one study, motor impairment predicted the cognitive performance not just among people with schizophrenia, but also among their unaffected first-degree relatives^19^. Cognitive deficits are similarly evident also in groups with psychosis risk symptoms and among unaffected relatives^25^. Abnormal motor performance associated with impaired cognition across multiple domains in individuals with high psychosis risk^26^.

As cognitive deficits are among the symptoms that severely interfere with daily functioning in psychosis, it is important to understand factors that may influence cognitive performance. Motor symptoms have been a part of the psychosis concept since Kraepelin, however, here we investigated the implications of mild EP symptoms instead of more severe abnormal psychomotor behaviors. We assessed EP symptoms with the Simpson-Angus Scale^27^, which measures motor effects such as tremor and stiffness. We investigated how EP symptoms associated with performance in cognitive testing during the first year after the onset of FEP. We took into account the daily dose equivalents of antipsychotic medications and the severity of psychosis symptoms, since antipsychotic medication may be partially or wholly responsible for these associations.

A possible mechanism explaining the associations between EP symptoms and cognition is that performance in cognitive tasks is affected by motor disturbances caused by the EP symptoms^5^. EP symptoms such as psychomotor retardation are associated to prolonged reaction times resulting in poor performance in cognitive tasks dependent of motor activity^18^. Therefore, we sought to determine whether current EP symptoms associated with deficits in motor and speed-reliant tasks, with the main hypothesis that even mild EP symptoms could impair performance in these, but not other types of tasks.

Additionally, based on previous results on the predictive value of EP symptoms on disease severity^10,22^, we tested a secondary hypothesis of whether EP symptoms act as a prognostic marker for cognitive symptoms by testing for longitudinal associations between baseline EP symptoms and cognitive performance a year later. Investigating cognitive correlates of minor motor abnormalities in FEP in a follow-up setting separates this work from many previous studies.

## Results

### Participants and their cognitive performance

The participants with cognitive data included 256 persons: 113 with FEP (71 from the Helsinki site and 42 from the Turku site), as well as 143 control participants (62 from Helsinki and 81 from Turku). Social cognition data were available for 66 FEP participants and 62 controls from Helsinki. Of the FEP group, 82% were diagnosed with nonaffective psychotic disorder (ICD diagnosis codes F20–29) and 18% with affective psychotic disorder (psychotic depression or bipolar disorder). The most common antipsychotics among patients were risperidone (36%), olanzapine (34%), and quetiapine (20%) at baseline, and olanzapine (17%), aripiprazole (14%), risperidone (13%), and quetiapine (11%) at follow-up. Supplementary Table 1 shows the participant demographics divided by group and by research site. The FEP and control groups, when combined across sites, did not differ in terms of age (*p* = 0.238) or gender (*p* = 0.140). FEP participants from the two sites did not differ in age (*p* = 0.891), gender (*p* = 0.213), or the symptom sum scores at either time point (*p* ≥ 0.085). In the controls, there was a difference between the sites in the gender distribution (*p* = 0.001) but not in age. Participants attending or not attending follow-up did not differ in terms of baseline cognition (*g* factor *p* = 0.090), EP symptoms (*p* = 0.321), or negative symptoms (*p* = 0.371), but those attending follow-up had lower baseline positive symptoms (*p* = 0.023).

Cognitive results can be seen in Table 1 and Supplementary Table 1. Unsurprisingly, the baseline cognitive performance of the controls was higher than in the FEP group (common language effect sizes (CL) = 0.14, *p* < 0.001). Participants with FEP from the two sites did not differ in terms of baseline or follow-up cognitive factors.

**Table 1:** FEP participants with demographic and clinical information, divided by baseline EP symptom groups. Mean (sd), range; or count (percent).

Measure	All FEP, *n* = 113		Baseline EP symptoms, *n* = 108		
		*n*	No EP symptoms, *n* = 65 (60.2%)	Any EP symptoms, *n* = 43 (39.8%)	Group difference^a^
**Baseline**					
Age	26.1 (5.6), 18.2–41.1	113	26.3 (5.4), 18.3–39.1	25.9 (5.9), 18.2–41.1	0.496
Females	48 (42.5%)	113	32 (49.2%)	14 (32.6%)	0.086
*g* factor	−1.1 (0.8), −3.0–0.8	109	−0.9 (0.7), −2.6–0.6	−1.3 (0.8), −3.0–0.8	**0.010**
Verbal factor	−1.0 (0.8), −2.8–0.9	109	−0.8 (0.8), −2.6–0.7	−1.1 (0.8), −2.8–0.9	**0.026**
Visuomotor factor	−1.1 (0.8), −2.9–0.7	109	−0.9 (0.7), −2.7–0.5	−1.3 (0.8), −2.9–0.7	**0.005**
Social cognition factor	−0.8 (1.7), −4.7–2.4	66	−0.6 (1.7), −4.2–2.4	−1.0 (1.7), −4.7–2.2	0.312
Nonaffective psychotic disorder	93 (82.3%)	113	54 (83.1%)	35 (81.4%)	0.741
CPZE	326.4 (239.2), 19–1320	100	322.0 (261.7), 18.8–1320.0	334.6 (210.0), 50.0–900.0	0.252
Positive symptoms^b^	8.3 (3.5), 3–16	110	8.3 (3.5), 3–16	8.4 (3.5), 3–14	0.803
Negative symptoms^c^	2.0 (1.2), 1–6	110	1.7 (0.9), 1–4	2.2 (1.3), 1–5	0.074
**Follow-up**					
Any EP symptoms	30 (37.5%)	80	11 (22.0%)	19 (63.3%)	**<0.001**
*g* factor	−0.9 (0.9), −3.3–1.1	64	−0.6 (0.8), −2.3–0.9	−1.3 (1.0), −3.3–1.1	**0.004**
Verbal factor	−1.0 (0.8), −3.2–0.8	64	−0.7 (0.7), −2.5–0.4	−1.4 (0.9), −3.2–0.8	**0.002**
Visuomotor factor	−0.7 (0.9), −2.9–1.4	64	−0.5 (0.7), −2.0–1.4	−1.0 (1.0), −2.9–1.2	**0.006**
CPZE	316.3 (222.6), 19–1050	64	316.4 (214.6), 30.0–850.0	316.2 (236.4), 18.8–1050.0	0.126
Antipsychotics	64 (78.0%)	82	36 (70.6%)	28 (90.3%)	**0.036**
Positive symptoms	4.6 (2.5), 3–13	81	4.5 (2.5), 3–13	4.8 (2.5), 3–11	0.296
Negative symptoms	1.9 (1.0), 1–4	81	1.8 (1.0), 1–4	2.0 (1.1), 1–4	0.366

CPZE, chlorpromazine equivalent.

EP, extrapyramidal.

FEP, first-episode psychosis.

^a^*p*-value, Mann-Whitney or Pearson’s Χ^2^ test.

^b^Sum of BPRS (or PANSS) Hallucinations, Delusions, and Conceptual disorganization.

^c^BPRS (or PANSS) blunted affect.

Rank-order correlations between measures in the FEP group are shown in Supplementary Table 2. Baseline cognitive factors were not correlated with chlorpromazine equivalents (CPZE) levels at either time point. However, higher follow-up performance was correlated with lower follow-up CPZE levels (r with the *g* factor = −0.34, *p* = 0.006).

As for the associations between cognition and symptoms, at baseline, negative symptoms correlated negatively with all cognitive factors (r with the *g* factor = −0.23, *p* = 0.017). At follow-up, neurocognitive factors had significant negative correlations with both positive and negative symptom levels.

CPZE levels and symptom levels were not associated at baseline, but at follow-up, CPZE levels were correlated with symptom levels, especially higher positive symptoms (Supplementary Table 2).

### Extrapyramidal symptoms

Single EP symptom scores are presented in Supplementary Table 3. Three control subjects (2%) were rated with some EP symptoms (scored as 1) at both baseline and follow-up, while the other controls were rated as having no EP symptoms. EP symptoms were present in 40% of the FEP participants at baseline. Table 1 shows the FEP participants divided into those with or without any baseline EP symptoms. At follow-up, 38% FEP participants had at least one EP symptom, the symptoms being rather stable between the time points (*p* < 0.001 in Χ^2^ test for having EP symptoms at the two time points).

EP symptoms were not associated with age or gender in the FEP group (Table 1). EP symptoms also did not differ between research sites (Χ^2^ *p* > 0.05). Although baseline EP symptoms did not differ between diagnostic groups, at follow-up EP symptoms were more common among participants with nonaffective psychosis (Χ^2^ *p* = 0.021).

The presence of baseline EP symptoms was unrelated to symptom severity. Negative symptom levels tended to be higher in patients with any baseline EP symptoms (mean 2.2) than in those without them (mean 1.7) but the difference did not meet statistical significance (*p* = 0.074; Table 1).

At follow-up, having EP symptoms was associated with more severe negative symptoms (CL = 0.53, *p* = 0.001), but not with positive symptoms.

Furthermore, EP symptoms were not associated with CPZE at baseline (Table 1).

Follow-up EP symptoms and CPZE were not associated either (*p* = 0.765). Of the patients with follow-up EP symptom data, out of the 60 patients still using antipsychotics, 43% presented with EP symptoms, compared to 24% of the 17 not using antipsychotics (Χ^2^ *p* = 0.139).

### Extrapyramidal symptoms and cognition

**Fig. 1:**
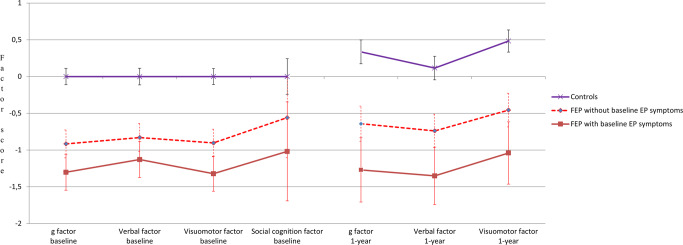
Baseline and follow-up cognitive factor scores (with 95% confidence intervals) in the study groups.

As can be seen in Table 1, baseline EP symptoms of FEP participants were associated with lower baseline and follow-up neurocognitive performance, both at the composite neurocognitive score level and considering the two neurocognitive domains separately. Baseline EP symptoms were not associated with baseline social cognition. Figure 1 presents cognitive factors longitudinally in FEP with or without baseline EP symptoms as well as in controls.

These associations were not significant among those diagnosed with affective psychosis (all *p* ≥ 0.240). In the nonaffective group, baseline EP symptoms were associated with lower baseline *g* factor (*p* = 0.008) and verbal (*p* = 0.012) and visuomotor performance (*p* = 0.006) but not social cognition (*p* = 0.126), as well as with the follow-up *g* factor (*p* = 0.009), verbal (*p* = 0.009), and visuomotor (*p* = 0.013) performance.

In addition, in the whole FEP group, having EP symptoms at follow-up was associated with lower follow-up visuomotor performance (CL = 0.33, *p* = 0.028), but not with follow-up general neurocognition (*p* = 0.066) or verbal performance (*p* = 0.110). None of these associations reached statistical significance if the FEP group was divided into diagnostic groups.

The associations of EP symptoms and single task scores can be seen in Supplementary Table 4. The strongest associations were found between baseline EP symptoms and the baseline Trail Making and Digit Symbol tests, and the follow-up Verbal Fluency and Digit Symbol tests. Having EP symptoms at follow-up was associated with weaker performance in Trail Making A and Spatial Span at baseline, and with weaker Digit Symbol performance at follow-up testing.

In linear regression models, when controlling for baseline positive and negative symptoms and CPZE (which were not significant predictors), having baseline EP symptoms was associated with a lower baseline *g* factor (B = −0.3 (95% CI −0.7, −0.02), β = −0.2, *p* = 0.037; model R^2^ = 0.08, adj. R^2^ = 0.04). Looking at the two neurocognitive domains separately, with the same predictors, baseline EP symptoms were associated with lower baseline visuomotor performance (B = −0.4 (95% CI −0.7, −0.1), β = −0.2, p = .021). However, baseline EP symptoms did not significantly associate with verbal performance (B = −0.3 (95% CI −0.6, 0.1), β = −0.2, *p* = 0.096) or social cognition (B = −0.2 (95% CI −1.1, 0.6), β = −0.1, *p* = 0.561).

In regression models predicting follow-up neurocognition with baseline EP symptoms, having EP symptoms at illness onset predicted a lower *g* factor a year later (B = −0.6 (95% CI −1.0, −0.1), β = −0.3, *p* = 0.013; model *R*^2^ = 0.19, adj. *R*^2^ = 0.14), again controlling for the same baseline variables. Using the two neurocognitive factors, baseline EP symptoms predicted both follow-up Verbal (B = −0.6 (95% CI −1.0, −0.2), β = −0.3, *p* = 0.007) and follow-up Visuomotor performance (B = −0.5 (95% CI −1.0, −0.1), β = −0.3, *p* = 0.017).

Finally, follow-up EP symptoms were not a significant predictor of follow-up *g* score (B = −0.1 (95% CI −0.6, 0.4), β = −0.1, *p* = 0.659) or Verbal or Visuomotor domains.

## Discussion

We investigated extrapyramidal signs as predictors of performance in cognitive testing in the early stages of psychotic illness: soon after entering to treatment and again 9–18 months later (mean follow-up time one year). Gait, elbow rigidity, and tremor scores of the Simpson-Angus Scale were used for assessment of EP symptoms. Forty percent of the individuals with a recent FEP had mild levels of EP symptoms and none had more severe EP symptoms. Before controlling for confounding factors, having EP symptoms at baseline associated cross-sectionally with baseline neurocognitive impairment, as well as longitudinally with follow-up neurocognitive impairment. A year later, cross-sectional associations between EP symptoms and visuomotor neurocognition (but not global or verbal cognition) were found. No associations between EP symptoms and social cognition were detected.

In regression models, we found that soon after the onset of psychosis, EP symptoms associated with neurocognitive impairment. The association was not explained by severity of psychopathology or antipsychotic dose. At the neurocognitive domain level, baseline EP symptoms predicted baseline visuomotor performance, but the association between EP symptoms and verbal performance was not statistically significant when positive and negative symptoms and antipsychotic medication were controlled for. Looking at longitudinal associations, baseline EP symptoms predicted follow-up neurocognition at the composite *g* factor level, and both the verbal and visuomotor domains separately. There were no cross-sectional associations between EP symptoms and cognition at follow-up when controlling for symptom severity and medication.

We had hypothesized that motoric slowing and stiffness would specifically affect processing speed but not necessarily the other cognitive domains. The tasks with speed limits (Verbal Fluency, Trail Making, and Digit Symbol), where slowing down clearly affects performance, were indeed significantly associated with EP symptoms on the single task level (Supplementary Table 4). Our results therefore suggest that cognitive speed may be most influenced by EP symptoms. This is in line with previous results by Fervaha and colleagues^18^, who found that the association between EP symptoms and cognition could be explained by motor speed, and concluded that EP symptoms affect *performance on cognitive tasks* rather than *core neurocognitive abilities* per se. Another study found that processing speed largely explained the cognitive decline in people with first-episode schizophrenia^28^, highlighting the importance of processing speed on cognition. In addition, the role of deficits in executive functioning, such as initiation and planning, is crucial in the neurocognitive testing situation, and can be linked to response inhibition or delays.

Social cognition was assessed in a subsample and only in one time point; those results are thus preliminary. However, our finding of no significant association between EP symptoms and social cognition is in line with a recent study by Monteleone and colleagues^5^ who reported that although EP symptoms associated with impaired social cognition in persons with schizophrenia, the association was not direct, but rather mediated by other factors, such as neurocognition.

Most of the FEP participants in this study were diagnosed with nonaffective psychotic disorder. Mild EP symptoms could be seen in both diagnostic groups, but more often in those with nonaffective psychosis. The associations between EP symptoms and neurocognitive performance were seen only in the nonaffective group and they were not statistically significant among those diagnosed with psychotic depression or bipolar disorder; however, this could have resulted from lack of power due to smaller group size.

Controlling for positive and negative symptom severity did not change our results, suggesting that the association between EP symptoms and neurocognition was not explained by these clinical features. It should be noted that neurocognitive tests were not performed until major psychotic symptoms were resolved. Blunted affect, a negative symptom, may be associated with the EP symptom of inexpressiveness, and EP symptoms may affect both negative symptom ratings and cognitive performance. We found that having EP symptoms at follow-up associated with more severe blunted affect, and not with positive symptom severity. The overlap of negative symptoms and cognitive deficits is a common finding^13^, as we have also previously found in our sample when predicting follow-up outcomes^29^. In a recent study, it was found that symptom level, antipsychotics, and especially level of functioning accounted for a significant portion of the cognitive impairment in individuals with psychotic disorders^30^. Thus, the association between cognition and negative symptoms could partly be explained by EP symptoms. Overlap may appear between EP symptoms and negative symptoms also in the sense that motor abnormalities are linked to reduced motor activity^31^.

We found that EP symptoms were not associated with antipsychotic medication dosage, as assessed by CPZE. Further, mild stiffness, tremor, or slowed movement could also be observed among those not using, or even naïve to, antipsychotic medication. Controlling for CPZE did not explain the association between EP symptoms and neurocognition. Although antipsychotic medication has been considered to affect cognitive processes, such as processing speed, and increase levels of certain negative symptoms, such as anhedonia and apathy, more research to elucidate the association between cognitive performance and antipsychotic medication is still needed^17^. Antipsychotics are used to alleviate positive symptoms and they are also associated with better cognitive level, but on the other hand, they can lead to EP symptoms and psychomotor slowing, highlighting the importance of finding the optimal level of antipsychotic treatment. There may also be differences between antipsychotic types on motor abnormalities, the effects varying from antipsychotics deteriorating motor abnormalities to improving preexisting abnormalities^32^. Our results supported the hypothesis of EP symptoms partly reflecting central neurobiological processes of psychotic disorders in addition to being antipsychotic-induced side effects^8,24^.

It should be noted that although both EP symptoms and neurocognitive performance were rather stable, after one year of follow-up the EP symptoms no longer predicted neurocognitive performance, and the reason for this remains unclear. Many confounding variables may affect cognition and its association with EP symptoms at follow-up, however, such as clinical factors, long-term use of antipsychotic medications, rehabilitation, and treatment, or attrition from the study.

### Strengths and limitations

To our knowledge, only a few studies^22^ have investigated the associations between EP symptoms and cognition longitudinally. Here individuals who had experienced their first psychotic episode were followed up for 9–18 months. Only three prototypical EP symptoms were evaluated at both sites but combining data from these two independent sites offered a larger sample. Assessment of EP symptoms was not blinded in terms of clinical information and medication use, and bias in their scoring cannot be completely ruled out. Assessing neurocognitive performance was limited to tasks administered at both sites, but factor models were used to quantify not only global neurocognition but also to differentiate between two neurocognitive domains, namely verbal and visuomotor performance, and social cognition could be analyzed in a smaller subsample. In our analyses, we took into account positive and negative symptom levels as well as antipsychotic medication dose. However the employed defined daily dose (DDD) method has its limitations, because DDDs have not been developed for the purpose of determining dose equivalence of dopamine receptor blockade^33^.

Whereas our interest was in the milder level of EP symptoms, previous works have typically studied the effect of more severe EP symptoms, for example, the use of anticholinergic medication to treat iatrogenic EP symptoms. Anticholinergic medication has been reported to associate with long-term cognitive impairment^34–37^. In the current study, anticholinergic medication was not controlled for, as such medication is very rarely used in Finland. On the other hand, some antipsychotics have anticholinergic effects which may affect cognition. In addition, although other medications, such as benzodiazepines, are not known to induce EP symptoms, they may affect cognitive performance.

## Conclusions

Mild EP symptoms were common among young adults with FEP, and not merely as side-effects of antipsychotic medication, but also as a psychosis symptom dimension. Even mild EP symptoms predicted poorer neurocognitive performance at illness onset, and independently from symptom severity or antipsychotic dose. EP symptoms may slow the person down and cause motor coordination difficulties in cognitive testing, but they might also reflect a more ubiquitous state of dopaminergic modulation affecting subcortical cognitive processing. Studying cognitive domains unaffected by psychomotor slowing could further elucidate the nature of these associations.

The associations between EP symptoms at illness onset and poorer neurocognitive performance a year later also cannot be explained by the effect of EP symptoms during the testing situation. Some earlier FEP studies have found that EP symptoms at illness onset may be markers of later neurocognitive impairment^22^ or clinical outcome^10,38^, irrespective for antipsychotic treatment. This is possibly related to dopamine system dysregulation^9^, or a disturbance in the cortical-striatal-thalamocortical neuronal network^39^. Further studies are needed to verify whether EP symptoms at treatment onset predict impaired neurocognitive outcome.

## Methods

### Participants and study protocol

Participants were recruited from two geographically distinct Finnish sites, Helsinki and Turku, both including young adults with first psychiatric treatment contact for affective and non-affective psychosis^40^. The participants were recruited from hospitals and outpatient clinics in 2010–2017 and in both sites interviewed with the Brief Psychiatric Rating Scale, Expanded version 4.0 (BPRS)^41^ as soon as possible after they had commenced treatment and were able to provide informed consent, as judged by the treating personnel. Of the FEP participants in Turku, 32 persons were interviewed with the Positive and Negative Syndrome Scale (PANSS)^42^ instead. Both sites used the Structured Clinical Interview for the DSM-IV, Research Version (SCID-I/P)^43^, with trained research staff conducting the interviews. Diagnoses were set by a senior psychiatrist based on SCID as well as medical records from received mental health treatment. As a criterion for inclusion in Helsinki, psychosis was defined as a score ≥ 4 (moderate or higher) in BPRS unusual thought content (delusions) or hallucinations. In Turku, the inclusion criterion was a psychotic disorder as defined by the Structured Interview for Prodromal Syndromes 5.0 Presence of Psychotic Symptoms criteria^44^, complemented by medical records. Exclusion criteria at both sites were substance-induced psychoses and psychotic disorders due to a general medical condition.

In addition, age- and gender-matched control participants from the same catchment areas were recruited through the Finnish Population Information System. The exclusion criteria were psychotic disorder, conditions preventing MRI, and chronic neurological or endocrinological diseases, but other mental health problems were allowed. Controls were assessed with the same measures as the FEP group.

The Helsinki Early Psychosis Study protocol included one meeting as soon as possible after the treatment had started (baseline) and follow-ups after two and twelve months. The cognitive testing was performed both at two months (in order to avoid testing in the most acute phase of the illness; referred here as the baseline cognitive testing) and twelve months^29^. The controls were assessed at baseline and again one year later. The Turku Early Psychosis Study included baseline and 9–12 month assessments for FEP and control participants^45^. In the whole FEP group used here, the follow-time varied between 272–539 days, mean ± SD 369 ± 47 days.

The participants gave written informed consent to participation. The study protocols were approved by the Ethics Committees of the Hospital Districts of Helsinki and Uusimaa and Southwest Finland, and by the institutional review boards of the Finnish Institute for Health and Welfare and the University of Helsinki. The study was carried out in accordance with the sixth version of the Declaration of Helsinki^46^.

### Cognitive assessment

Cognitive testing was administered by a psychologist at two time points to both FEP and control participants. In the present study, we use data for those tasks which were in use at both sites. These include measures from the Wechsler Adult Intelligence Scale, Third Edition (Vocabulary, Digit Symbol)^47^ and the Wechsler Memory Scale, Third Edition (Letter-Number Sequencing, Spatial Span)^48^, as well as the Trail Making Test^49^, and the Verbal Fluency test (semantic and phonemic)^50^.

To summarize baseline neurocognitive performance, a one-dimensional *g* factor model was estimated with Mplus 8.1^51^. In addition, we used a confirmatory two-dimensional factor model separating the correlated Verbal and Visuomotor factors, which has been presented in our previous work^40^. Supplementary Table 5 presents the loadings of the factor models.

For the follow-up cognition, factor scores were calculated using the baseline models with the same parameters, instead of estimating the models again. All the same tasks were used except for Vocabulary, which was not included at follow-up. The model descriptors of baseline and follow-up neurocognitive models are presented in Supplementary Table 6.

In addition, but only at the Helsinki site as part of the first testing, the theory of mind domain of social cognition was assessed with the Hinting task^52^. We have previously obtained a one-dimensional factor solution of the Hinting task^53^, taking into account the varying difficulty level and relevance of the task items, and the factor scores were used in the analyses as a measure of social cognition.

The cognitive factor scores were transformed into age- and gender-corrected residuals based on the performance of controls at baseline, to be used in all analyses.

### Extrapyramidal symptoms

EP symptoms were evaluated using shortened versions of the Simpson-Angus Scale^27^ in each study phase. On this scale, ratings from 0 to 4 are used, with 0 indicating “normal” motor functions, 1 indicating mild impairment, and higher scores increasingly severe impairment. Raters were trained research staff who also conducted clinical interviews. In Helsinki, five items were used: gait, arm dropping, elbow rigidity, leg pendulousness, and tremor. In Turku, items gait, elbow rigidity, and tremor were included. Here, we used the three items common to both sites: gait, elbow rigidity, and tremor. We divided the participants into those having 0 points on all three items and those having any EP symptoms. Supplementary Table 3 shows the ratings for all available items in the sites, including the 2-month EP scores from the Helsinki site, which were otherwise not used in the current analyses.

### Other measures

Similarly as in our previous work combining FEP participants from the two sites^40^, we used symptom equivalents for the FEP subgroup interviewed with PANSS instead of BPRS. We considered PANSS item P3 Hallucinatory behavior to correspond to BPRS item 10 Hallucinations, P1 Delusions in PANSS to correspond to item 11 Unusual thought content in BPRS, PANSS P2 Conceptual disorganization to correspond to BPRS item 15 Conceptual disorganization, and PANSS N1 Blunted Affect to correspond to BPRS item 16 Blunted affect.

At both time points, positive psychotic symptoms were calculated as the sum of hallucinations, unusual thought content, and conceptual disorganization item scores, while blunted affect was used to assess negative symptoms.

Information on medication was collected from interviews and medical records. The DDD based CPZE^33^ are reported.

### Statistical analyses

For statistical analyses we employed IBM SPSS Statistics for Windows, version 27^54^. The limit for statistical significance was *p* < 0.05. The age- and gender-corrected factor score residuals for the composite *g* factor, the two-dimensional neurocognitive factors, and the social cognition factor were used in the analyses. We also looked at neurocognitive correlates of EP symptoms on a single task score level.

The participant groups were compared with Pearson’s Χ^2^ or Mann-Whitney *U* tests. Spearman rank-order correlations (r) were used to examine the associations between continuous variables of interest, such as cognitive factor scores, symptom levels, and CPZE levels. To adjust for multiple comparisons in Supplementary Table 2, false discovery rate correction was calculated using the Benjamini–Hochberg procedure^55^.

Cross-sectional associations between EP symptoms and cognition at both time points were calculated separately with the Mann-Whitney *U* test, to see whether any associations would replicate (primary analyses), and then longitudinally between baseline EP symptoms and follow-up cognition (secondary analyses). These associations were investigated also separately in the two diagnostic groups. Common language effect sizes (CL) were calculated from Mann-Whitney values as *U*/n_1_ × n_2_.

Linear regression models were estimated in the FEP group, controlling for antipsychotic medication (CPZE), positive symptoms, and negative symptoms. The models predicted (1) baseline cognition with baseline predictors (baseline EP symptoms and other symptoms, and baseline medication) (2) follow-up cognition with the same baseline predictors, and (3) follow-up cognition with follow-up predictors (follow-up EP symptoms and other symptoms, and follow-up medication). For these regression models, we report unstandardized B coefficients with 95% confidence intervals (CI) as well as R^2^ and adjusted R^2^ values. In addition, standardized β values are provided to allow comparability between models.

## Supplementary information

Lindgren Supplement

## Data Availability

Data are from the Helsinki Early Psychosis Study at the Finnish Institute for Health and Welfare and from the Turku Early Psychosis Study at the Hospital District of Southwest Finland. Sharing of the data is possible in research collaborations if it is in agreement with the consent given by the participants and with the General Data Protection Regulation (GDPR) and other applicable law. Collaborations require a separate agreement and local ethical committee approval.

## Code availability

No software was used in data collection.

No custom code was used in data analysis. The commercial statistical software packages used (SPSS 27 & Mplus 8.1) are mentioned in the article (with version numbers) and the analyses are described in sufficient detail to reproduce the results without syntax/scripts provided.
