# Supplementary material for: Extrapyramidal symptoms predict cognitive performance after first-episode psychosis
Source: Schizophrenia (Heidelb). 2022 Aug 4;8(1):64. doi: 10.1038/s41537-022-00270-8 (PMC9352759; doi:10.1038/s41537-022-00270-8)
Supplement: Supplementary file 1 — Lindgren Supplement [file 41537_2022_270_MOESM1_ESM.pdf]

Supplementary Table 1. Participants with demographic and clinical information, divided by patient/control group and by research site. Mean (sd), range; or count (percent).

	FEP			Controls		
Total n=256	All, n=113	Helsinki, n=71	Turku, n=42	All, n=143	Helsinki, n=62	Turku, n=81
**Baseline**						
Age	26.1 (5.6), 18.2–41.1	26.1 (5.6), 18.2–41.1	26.2 (5.6), 19.0–39.0	27.3 (6.7), 19.0–56.0	26.4 (5.9), 19.1–43.9	28.0 (7.2), 19.0–56.0
Females	48 (42.5%)	27 (38%)	21 (50.0%)	74 (51.7%)	22 (35.5%)	52 (64.2%)
EP symptom sum [1]	0.7 (1.0), 0–4	0.7 (1.0), 0–4	0.6 (1.0), 0–4	0.02 (0.1), 0–1	0	0.04 (0.2), 0–1
Any EP symptoms	43 (39.8%)	30 (42.3%)	13 (35.1%)	3 (2.1%)	0	3 (3.7%)
g factor	-1.1 (0.8), -3.0–0.8	-1.1 (0.8), -3.0– 0.8	-1.2 (0.7), -2.5–0.4	0.0 (0.7), -2.2–2.2	-0.1 (0.7), -1.6–2.2	0.1 (0.6), -2.2–1.5
Verbal factor	-1.0 (0.8), -2.8–0.9	-1.0 (0.8), -2.8–0.9	-1.0 (0.7), -2.4–0.7	-0.0 (0.7), -2.3–2.1	-0.1 (0.7), -1.4–2.1	0.1 (0.6), -2.3–1.4
Visuomotor factor	-1.1 (0.8), -2.9–0.7	-1.0 (0.8), -2.9–0.7	-1.2 (0.7), -2.5–0.4	0.0 (0.7), -2.0–2.2	0.0 (0.7), -1.6–2.2	0.0 (0.6), -2.0–1.5
Social cognition factor	-	-0.8 (1.7), -4.7–2.4	-	-	0.0 (1.0), -2.5–1.7	-
Nonaffective psychotic disorder	93 (82.3%)	61 (85.9%)	32 (76.2%)	-	-	-
CPZE	326.4 (239.2), 19–1320	360.7 (271.9), 19–1320	259.9 (138.1), 60–600	-	-	-
Positive symptoms [2]	8.3 (3.5), 3–16	8.5 (3.5), 3–15	8.1 (3.4), 3–16	-	-	-
Negative symptoms [3]	2.0 (1.2), 1–6	1.8 (1.1), 1–5	2.3 (1.4), 1–6	-	-	-
**Follow-up**						
EP symptom sum	0.6 (0.9), 0–3	0.7 (0.9), 0–3	0.5 (0.9), 0–3	0.03 (0.2), 0–1	0.1 (0.3), 0–1	0
Any EP symptoms	30 (37.5%)	24 (42.1%)	6 (26.1%)	3 (3.2%)	3 (6.8%)	0
g factor	-0.9 (0.9), -3.3–1.1	-0.8 (1.0), -2.9–1.1	-1.0 (0.8), -3.3–0.0	0.3 (0.8), -1.6–2.4	0.1 (0.8), -1.6–2.4	0.5 (0.7), -0.9–2.1
Verbal factor	-1.0 (0.8), -3.2–0.8	-0.9 (0.9), -2.8–0.8	-1.1 (0.8), -3.2–0.4	0.1 (0.8), -1.6–2.2	-0.1 (0.8), -1.6–2.0	0.3 (0.7), -1.2–2.2
Visuomotor factor	-0.7 (0.9), -2.9–1.4	-0.6 (0.9), -2.6–1.4	-0.9 (0.7), -2.9–0.0	0.5 (0.7), -1.4–2.4	0.3 (0.8), -1.4–2.4	0.6 (0.7), -0.7–2.0
CPZE	316.3 (222.6), 19–1050	330.3 (228.7), 19–1050	280.4 (208.1), 30–850	-	-	-
Antipsychotics	64 (78.0%)	46 (79.3%)	18 (75.0%)	-	-	-
Positive symptoms	4.6 (2.5), 3–13	4.5 (2.3), 3–13	4.8 (2.8), 3–11	-	-	-
Negative symptoms	1.9 (1.0), 1–4	1.8 (1.0), 1–4	2.0 (1.1), 1–4	-	-	-

CPZE, chlorpromazine equivalent
EP, extrapyramidal
FEP, first-episode psychosis
[1] Sum scores for items gait, elbow rigidity, and tremor; each rated on a scale of 0–4 (maximum total score 12).
[2] Sum of BPRS (or PANSS) Hallucinations, Delusions, and Conceptual disorganization.
[3] BPRS (or PANSS) blunted affect

Supplementary Table 2. Rank-order (Spearman) correlations between cognitive factors, medication, and symptom severity in the FEP group.

			Baseline cognition				Follow-up cognition			CPZE		Symptoms		
			g factor	Verbal factor	Visuo-motor	Social cognition	*g* factor	Verbal factor	Visuo-motor	Baseline CPZE	Follow-up CPZE	Baseline positive	Baseline negative	Follow-up positive
Baseline cognition	Verbal factor	r	**0.96**	1										
		p	**<0.001***											
	Visuomotor factor	r	**0.98**	**0.90**	1									
		p	**<0.001***	**<0.001***										
	Social cognition	r	**0.31**	**0.36**	**0.25**	1								
		p	**0.012***	**0.003***	**0.045**									
Follow-up cognition	*g* factor	r	**0.73**	**0.70**	**0.71**	**0.49**	1							
		p	**<0.001***	**<0.001***	**<0.001***	**0.002***								
	Verbal factor	r	**0.68**	**0.68**	**0.64**	**0.46**	**0.96**	1						
		p	**<0.001***	**<0.001***	**<0.001***	**0.003***	**<0.001***							
	Visuomotor factor	r	**0.73**	**0.69**	**0.74**	**0.51**	**0.98**	**0.91**	1					
		p	**<0.001***	**<0.001***	**<0.001***	**0.001***	**<0.001***	**<0.001***						
CPZE	Baseline CPZE	r	-0.08	-0.10	-0.06	-0.10	-0.02	-0.01	-0.01	1				
		p	0.384	0.316	0.540	0.422	0.882	0.919	0.938					
	Follow-up CPZE	r	-0.14	-0.17	-0.10	-0.24	**-0.34**	**-0.38**	**-0.30**	**0.48**	1			
		p	0.213	0.149	0.400	0.087	**0.006***	**0.002***	**0.020***	**<0.001***				
Symptoms	Baseline positive	r	0.01	0.02	0.02	-0.10	-0.18	-0.19	-0.18	0.06	0.17	1		
		p	0.924	0.833	0.857	0.417	0.165	0.134	0.158	0.547	0.132			
	Baseline negative	r	**-0.23**	**-0.20**	**-0.25**	**-0.28**	**-0.32**	**-0.28**	**-0.32**	-0.05	0.22	0.07	1	
		p	**0.017***	**0.045**	**0.009***	**0.024***	**0.011***	**0.023***	**0.009***	0.602	0.051	0.487		
	Follow-up positive	r	-0.07	-0.10	-0.05	0.15	**-0.28**	**-0.31**	-0.24	0.00	**0.36**	**0.40**	0.11	1
		p	0.532	0.396	0.663	0.294	**0.027**	**0.013***	0.060	0.998	**0.001***	**<0.001***	0.338	
	Follow-up negative	r	-0.17	-0.17	-0.16	**-0.31**	**-0.44**	**-0.46**	**-0.41**	-0.02	**0.23**	0.19	**0.29**	**0.40**
		p	0.143	0.139	0.170	**0.026**	**<0.001***	**<0.001***	**0.001***	0.867	**0.044**	0.091	**0.008***	**<0.001***

CPZE, chlorpromazine equivalent
r, correlation coefficient
p, significance level (p values <.005 are marked with bold font)
* Correlation is significant after adjusting for False Discovery Rate (p<0.028).

Supplementary Table 3. Single extrapyramidal symptoms in the FEP and control groups, divided by research site, rated on a scale of 0–4. Mean (sd), range.

	FEP			Controls		
	All	Helsinki	Turku	All	Helsinki	Turku
Baseline		n=71	n=37		n=61	n=81
Gait	0.3 (0.5), 0–2	0.3 (0.6), 0–2	0.2 (0.5), 0–2	0	0	0
Arm dropping	0.3 (0.6), 0–3	0.3 (0.6), 0–3	-	0.0 (0.1), 0–1	0.0 (0.1), 0–1	-
Elbow rigidity	0.2 (0.4), 0–2	0.2 (0.4), 0–2	0.2 (0.5), 0–2	0.0 (0.1), 0–1	0	0.0 (0.1), 0–1
Leg pendulousness	0.1 (0.5), 0–3	0.1 (0.5), 0–3	-	0	0	-
Tremor	0.2 (0.4), 0–2	0.2 (0.4), 0–2	0.2 (0.4), 0–1	0.0 (0.1), 0–1	0	0.0 (0.2), 0–1
2-month follow-up		n=64				
Gait	-	0.4 (0.6), 0–2	-	-	-	-
Arm dropping	-	0.2 (0.5), 0–2	-	-	-	-
Elbow rigidity	-	0.3 (0.5), 0–3	-	-	-	-
Leg pendulousness	-	0.2 (0.4), 0–2	-	-	-	-
Tremor	-	0.1 (0.3), 0–1	-	-	-	-
9–12-month follow-up		n=57	n=23		n=44	n=51
Gait	0.3 (0.6), 0–2	0.4 (0.6), 0–2	0.1 (0.5), 0–2	0.0 (0.2), 0–1	0.1 (0.3), 0–1	0
Arm dropping	0.3 (0.5), 0–2	0.3 (0.5), 0–2	-	0	0	-
Elbow rigidity	0.2 (0.4), 0–1	0.2 (0.4), 0–1	0.1 (0.3), 0–1	0	0	0
Leg pendulousness	0.1 (0.3), 0–1	0.1 (0.3), 0–1	-	0.0 (0.2), 0–1	0.0 (0.2), 0–1	-
Tremor	0.1 (0.3), 0–1	0.1 (0.3), 0–1	0.3 (0.4), 0–1	0	0	0

Supplementary Table 4. Associations between EP symptoms and raw scores of single cognitive tasks.

Task	EP symptoms baseline		EP symptoms follow-up	
Baseline tasks	Mann-Whitney test	Effect size % [1]	Mann-Whitney test	Effect size %
WMS-III Letter Number Sequencing	U=1191.0, p=0.734	48	U=650.0, p=0.929	49
Fluency animals + letter S	U=870.5, p=0.093	40	U=407.5, p=0.060	36
WAIS-III Vocabulary	U=1006.0, p=0.265	43	U=493.5, p=0.293	43
Trail Making A, Inverted	U=983.0, p=**0.034**	38	U=492.5, p=**0.043**	36
Trail Making B, Inverted	U=849.5, p=**0.019**	36	U=481.5, p=0.120	39
WMS-III Spatial Span total	U=1001.0, p=0.099	40	U=453.5, p=**0.024**	34
WAIS-III Digit Symbol	U=740.0 p=**0.001**	31	U=497.5, p=0.102	39
Follow-up tasks				
WMS-III Letter Number Sequencing	U=481.5, p=0.888	51	U=499.5, p=0.652	53
Fluency animals + letter S	U=209.5, p=**0.001**	24	U=328.5, p=0.140	39
Trail Making A, Inverted	U=381.5, p=0.207	40	U=383.5, p=0.231	41
Trail Making B, Inverted	U=393.5, p=0.342	43	U=381.0, p=0.278	42
WMS-III Spatial Span total	U=428.5, p=0.545	45	U=359.0, p=0.120	38
WAIS-III Digit Symbol	U=225.0, p=**0.001**	24	U=318.5, p=**0.047**	35

Note: All significant group differences (in bold) are in the direction of EP symptoms associating with worse neurocognition.
[1] Common language effect sizes calculated from Mann-Whitney values as U / n1 × n2.
The values are 0-100%, indicating the probability that in pairwise comparisons, a random participant without EP symptoms performs better in the task than a random participant with EP symptoms; 50% meaning no group difference and 100% meaning that the non-EP group always performs worse than the EP group.

Supplementary Table 5. Standardized factor loadings for the baseline cognitive models.

	Standardized factor loadings		
	Single-dimensional model	Two-dimensional model	
Task		Verbal factor	Visuomotor factor
WMS-III Letter Number Sequencing	0.60	0.60	
Verbal Fluency, animals	0.71	0.76	
Verbal Fluency, letter S	0.58	0.65	
WAIS-III Vocabulary	0.52	0.60	
Trail Making A (Inverted)	0.60		0.65
Trail Making B (Inverted)	0.70		0.74
WMS-III Spatial Span total	0.46		0.49
WAIS-III Digit Symbol	0.81		0.82

Supplementary Table 6 a. Neurocognitive model descriptors.

Index	Baseline		Follow-up	
	Single-dimensional model	Two-dimensional model	Single-dimensional model	Two-dimensional model
Comparative Fit Index (CFI)	0.89	0.94	0.80	0.92
Standardized Root Mean Square Residual (SRMR)	0.06	0.05	0.16	0.15
Factor determinacy	0.86	0.90, 0.90	0.94	0.89, 0.91

Supplementary Table 6 b. Unstandardized model parameters for the baseline cognitive models.

Task	Loadings		
	Single-dimensional model	Two-dimensional model	
		Verbal factor	Visuomotor factor
WMS-III Letter Number Sequencing	1.50	1.50	
Verbal Fluency, animals	4.59	4.96	
Verbal Fluency, letter S	3.28	3.70	
WAIS-III Vocabulary	4.81	5.56	
Trail Making A (Inverted)	7.38		8.02
Trail Making B (Inverted)	4.62		4.84
WMS-III Spatial Span total	1.40		13.63
WAIS-III Digit Symbol	13.51		1.50
